# A descriptive study of adrenal crises in adults with adrenal insufficiency: increased risk with age and in those with bacterial infections

**DOI:** 10.1186/1472-6823-14-79

**Published:** 2014-10-01

**Authors:** R Louise Rushworth, David J Torpy

**Affiliations:** School of Medicine, Sydney, The University of Notre Dame, Australia, 60 Oxford St, Darlinghurst, NSW 2010 Australia; Endocrine and Metabolic Unit, Royal Adelaide Hospital and Discipline of Medicine, University of Adelaide, Adelaide, SA Australia

**Keywords:** Adrenal crisis, Hypoadrenalism, Infection

## Abstract

**Background:**

An adrenal crisis (AC) is a major cause of morbidity in hypoadrenal patients. However, there is little information available on the incidence and underlying causes of AC.

**Methods:**

The aim of the present study was to describe the incidence of AC in New South Wales (NSW), Australia. Using a health department database, we selected de-identified data on all adults aged 20 years and over who were treated in any hospital in NSW between July1, 2000-June 30, 2011, with either a principal or secondary diagnosis of an AC. AC admission rates were calculated overall and within age categories. Frequencies of co-morbid diagnoses were analysed by age and sex groups. Poisson regression was used to assess the significance of the observed change in AC related admissions with age, while controlling for any secular trends by including year in the model. Chi sq tests were used to assess the differences in frequencies of categorical variables between groups.

**Results:**

824 patients received treatment for an AC in hospital, corresponding to 74.9 admissions/year. The majority (62.5%) of the patients were women and 52.8% were aged 60 years and over. Admission rates were significantly associated with increasing age (p < 0.0001). Patients in the 60–69, 70–79 and 80+ age groups had the highest average admission rates (24.3, 35.2 and 45.8 per million/year). A principal or secondary diagnosis of an infection was reported in 317 (38.5%) patients and infection was significantly associated with age (p < 0.0001) with older patients having the highest proportion of cases. The most frequent infections were pneumonia/lower respiratory tract infection in 85 (10.3%) cases and urinary tract infection (UTI) in 82 (10.0%) patients. Women experienced 78.0% of the reported UTIs. There were 125 patients (15.2%) with an AC and a record of gastroenteritis. Twenty-six (3.2%) patients died in hospital but, of these, only 4 deaths (0.9%) were recorded among the 467 patients with a principal diagnosis of an AC.

**Conclusions:**

The incidence of AC increases with age. Infections, especially bacterial infections, are associated with the incidence of ACs and this increases with age.

## Background

Adrenal insufficiency (AI) is a rare health problem, which is associated with significant morbidity and an increased risk of mortality [1, 2]. The prevalence of primary adrenal insufficiency (PAI) in Australia is not known but it is assumed to be approximately 100 cases per million, as it is in a number of other countries [3]. Secondary adrenal insufficiency (SAI), which is due to disease in the pituitary or hypothalamus, is thought to be more common than PAI by a factor of 2:1 [3–5]. Autoimmune disease is regarded as the most common underlying cause of Addison’s disease (AD) in Australia and, as autoimmune disease is more common in women, it is assumed that women comprise the majority of patients affected by AD [3]. Adrenal crises (ACs), which are acute alterations in physiology due to adrenal hormone deficiency, are a life-threatening consequence of AI [1, 2, 6]. They comprise symptoms such as vomiting and syncope and signs including hypotension and electrolyte abnormalities.

Adrenal steroid replacement therapy consists of a glucocorticoid (GC) (cortisone acetate or hydrocortisone) or a longer acting GC (prednisolone/prednisone or dexamethasone) and mineralocorticoid replacement therapy is also used in the majority of patients with PAI. In recent years, refinements have been made to the regimen of GC replacement therapy. These have been aimed at more closely reproducing the circadian pattern of cortisol secretion with the intention of minimizing the adverse effects of prolonged exposure to corticosteroids [6–8]. However, it is not known if lower dose, short-acting glucocorticoid replacement therapy may actually predispose patients with hypoadrenalism to ACs during illness by exposing them to periods of relatively profound hypocortisolaemia [9].

Prevention of an AC requires early recognition, usually by the patient, of a problem necessitating stress dosing followed, when appropriate, by prompt access to medical care and hospital attendance [3]. However, evidence suggests that many patients are ill-prepared to manage the decision-making required to avoid an AC in the event of intercurrent illness or injury [10]. Furthermore, there is evidence to indicate that clinical staff may be unfamiliar with the needs of patients with AI and may fail to institute adequate treatment in a timely manner [11, 12].

While there is evidence on the incidence and prevalence of AI in some countries, there is limited information on the causes and contributing factors of ACs. The aim of the study was to describe the incidence, demographic profile, patterns of morbidity and in-hospital mortality of patients aged 20 years and over, identified as having an AC, either during the hospital treatment episode or as the main reason for their admission, in all hospitals in New South Wales (NSW), Australia between 2000/1 and 2010/11.

## Methods

The State of NSW, Australia has a population of approximately 5 million adults aged 20 years and over (5,389,908 people in 2011) [13]. The age structure of the population is shown in Table 1. Clinical information on all admissions to public and private hospitals in NSW is reviewed and extracted from the health care record of each patient by staff trained in clinical coding. The documented diseases, injuries, complications, co-morbidities, and procedures noted in this record are then coded according to the International Classification of Diseases (ICD) 10 [14]. Each hospital conducts audits on the coded data, rectifies any errors and submits the data to the NSW Ministry of Health to form part of the Admitted Patient Data Collection (APDC). In addition to the auditing procedures conducted at the hospital level, the NSW Ministry of Health also subjects these data to regular quality assurance assessments.

For this study, we selected all admissions to NSW hospitals between July 1, 2000 and June 30, 2011 in which the principal diagnosis (main reason for the admission) or any secondary (co-morbid) diagnosis was coded as E27.2 (Addisonian Crisis). Each de-identified unit record included information on the year (according to the Australian financial year (July 1 to June 30)), hospital type (private or public), age, sex, mode of admission, mode of separation (departure from hospital, including in-hospital mortality), the length of hospital stay (LOS), principal diagnosis, all secondary diagnoses and any surgical procedures performed in an operating theatre during each patient’s admission.

An infection was classified as a principal or any secondary diagnosis in which the diagnosis was a disease or condition caused by an organism such as a urinary tract infection (UTI), pneumonia (excluding pneumonitis due to aspiration) or lower respiratory tract infection (LRTI), sepsis, or where an infectious agent was coded. A virus was identified as present when a specific viral agent was identified or where a diagnostic code had a viral agent included, such as “viral gastroenteritis”. A bacterial infection was identified when a bacterium as a cause of disease was coded in a diagnosis field or where a bacterial infection was identified but not named (B96.88- Other & unspecified bacterial agent cause of disease). Where a UTI (N39) was coded but there was no bacterial agent identified (23 cases) these cases were assumed to be bacterial in origin and were classified accordingly.

Gastroenteritis was identified by any code specifying gastroenteritis, whether viral, bacterial or non-infective. Colitis was also included in the gastroenteritis group but “nausea and vomiting” (R11) was not. “Pneumonia/ LRTI” was classified as any pneumonia, LRTI, acute bronchitis and chronic obstructive pulmonary disease (COPD) with mention of infection but excluded COPD, asthma exacerbation or bronchiectasis, where an infection was not mentioned.

For the purposes of analysis, age was reclassified into ten-year groups and the LOS was classified into 4 categories (0–1, 2–3, 4–6 and 7 or more days). Chi sq tests were used to assess the differences in frequencies of categorical variables between groups. Age-specific incidence rates were determined by dividing the number of AC admissions in each age category by the corresponding NSW population [13] in that age category for each year and this was then converted into a rate per million population per year. A mean rate for each age group for the study period was computed, together with 95% confidence intervals. Poisson regression was used to assess the significance of the observed change in rates of AC related admissions with age, while controlling for any secular trends by including year in the model. A value of p < 0.05 was considered significant.

The data were analysed using Excel (Microsoft. (2007) Microsoft Excel [computer software]. Redmond, Washington: Microsoft) and SPSS (IBM Corp. Released 2013. IBM SPSS Statistics for Windows, Version 22.0. Armonk, NY: IBM Corp.). The study was approved by the Human Resource Ethics Committee by the University of Notre Dame, Australia.

## Results

There were 824 hospital admissions during the study period in which an AC was recorded, corresponding to an overall rate of 74.9 admissions per year. The median age of patients was 61 years and there were more female (62.5%) than male patients (37.5%), (Table 1). The number of admissions increased with age up to the 70–79 year age group (Table 1). Over half (52.8%) the admissions with an AC were in the age category of 60 years and over. By comparison, only 26.9% of the comparable NSW population was aged 60 years or more (Table 1). Other demographic features of this patient group are outlined in Table 1.

**Table 1:** **Demographic characteristics of patients admitted with an AC, NSW, 2000/1 to 2010/11 (N = 824)**

Age group	Patients		NSW population ^#^		Rate/million/yr**
(yrs)	n	%	n	%	(95% CI)
20-29	87	10.6	1,021,381	18.9	8.3 (3.4, 15.8)
30-39	85	10.3	1,006,541	18.7	7.7 (3.4, 15.8)
40-49	93	11.3	996,337	18.5	9.0 (4.1, 17.1)
50-59	124	15.0	918,039	17.0	13.5 (7.7, 23.5)
60-69	153	18.6	719,740	13.4	24.3 (15.4, 35.7)
70-79	159	19.3	433,375	8.0	35.2 (24.4, 48.7)
80+	123	14.9	294,495	5.5	45.8 (33.7, 61.4)
Total	824		5,389,908		15.0 (8.4, 24.7)
**Sex**					
Males	309	37.5			
Admission*					
Emergency Dept	601	77.1			
Hospital transfer	69	8.8			
Doctor referral	60	7.7			
Other	50	6.4			
**Length of stay**					
0-1 days	155	18.8			
2-3 days	236	28.6			
4-6 days	185	22.5			
7 or more days	248	30.1			

^#^Population estimates for 2011 [11].

**Average rate over the study period.

*n = 780.

In 467 (56.7%) of the admissions, the patient’s principal diagnosis was an AC and 61 patients (7.4%) had an AC recorded as their only diagnosis. The proportion of admissions in which the principal diagnosis was an AC decreased with age, from over 68% in the youngest three age groups to 60.5% in the 50–59 year group, 56.2% in the 60–69 year group, 41.5% in the 70–79 year group and 43.1% in those patients aged 80 and above. An underlying cause of the AI was recorded in only 143 (17.4%) of the cases. There were 58 (7.0%) patients identified as having PAI and 48 (5.8%) with hypopituitarism and the remainder had other causes recorded (Table 2). Diabetes mellitus (DM) was reported in 178 (21.6%) of the patient admissions, 53 (6.4%) had Type 1 DM and a larger group of 125 (15.2%) had Type 2 DM. There were 26 (3.2%) deaths recorded for the whole patient group. However, among the 467 patients whose principal diagnosis was an AC, there were 4 (0.9%) deaths.

**Table 2:** **Frequency of diagnoses in 824 patients admitted with an AC, NSW, 2000/1-2010/11**

Diagnoses	n	%
Any infection	317	38.5
Bacterial infection	131	15.9
Viral infection	61	7.4
Gastroenteritis*	125	15.2
Pneumonia/LRTI	85	10.3
UTI	82	10.0
**Adrenal Causes of Hypoadrenalism**		
PAI	58	7.0
Congenital causes	20	2.4
Post procedural	4	0.5
Other and unspecified causes	9	1.1
Adrenal malignancy	5	0.6
Drug induced causes	4	0.5
Hypopituitarism	48	5.8
Type 1 Diabetes Mellitus	53	6.4
Type 2 Diabetes Mellitus	125	15.2
Fracture	22	2.7
Surgical procedure	45	5.5
In-hospital mortality	26	3.2

*Includes infective and non-infective.

The average admission rate for the study group was 15.0 (95% CI, 8.4, 24.7) admissions/million/year. While patients in the younger age groups had lower rates than average, those aged 60–69 years had a rate of 24.3 admissions/million/year, patients in the 70–79 year age group had a higher rate (35.2 admissions/million/year) and those patients age 80 years and over had the highest rate of 45.8 admissions/million/year. The average AC admission rates for each age group, together with the corresponding 95% CIs, are shown in Table 1. Poisson regression analysis demonstrated that the age group of the patient was significantly associated with the number of AC admissions (Wald Chi sq (6) =619.3, p < 0.0001).

**Figure 1:**
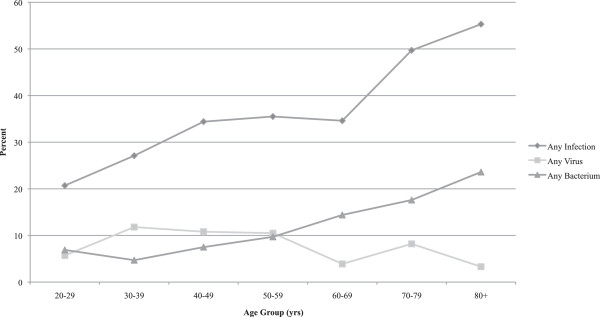
**Adrenal Crisis Admissions in NSW, 2000/1-2010/11.** Percentage with Any Infection, Virus or Bacterium by Age Group.

An infection was identified in either the principal or a secondary diagnosis field in 317 (38.5%) of patient admissions (Table 2). A record of infection was significantly associated with the age group of the patient (Chi sq (6) = 41.5, p < 0.0001) (Figure 1). One fifth (20.7%) of the patients in the youngest age group (20–29 years) were recorded as having an infection compared with 49.7% of the 70–79 years group and 55.3% of the oldest (80 and over) age group. Infections, which were recorded as bacterial in origin, were significantly more common in the study group than were infections due to viruses (131 (15.9%) and 61 (7.4%) respectively, z = 5.4, p < 0.01). Viral infections were not significantly associated with the age or sex of the patient. By comparison, bacterial infections were significantly associated with age (Chi sq (6) = 33.5, p < 0.0001) (Figure 1). Patients in the youngest age categories had the lowest levels of bacterial infection recorded (6.9% and 8.2% in the 20–29 and 30–39 year age groups respectively). In contrast, the group with the highest proportion of patients with a bacterial infection was the “80 and over” category, with 28.5% of the patients having a bacterial infection identified (Figure 1). 

**Figure 2:**
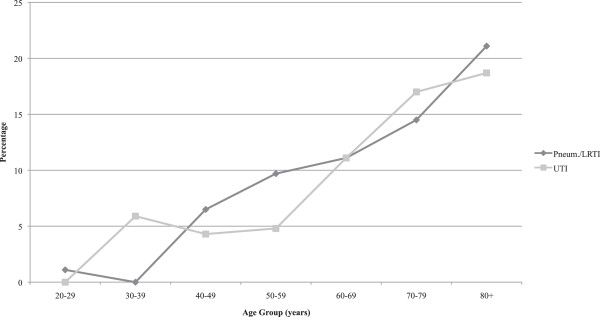
**Adrenal Crisis Admissions in NSW,2000/1-2010/11.** Percentage with Pneumonia/LRTI and UTI by Age and Sex.

There were 125 cases (15.2%) of gastroenteritis identified and of these only 27 (21.6%) were classified as being viral in origin. There was no significant association between the age and sex of the patient and the incidence of gastroenteritis. By comparison, pneumonia/LRTI was recorded in 85 (10.3%) of the patients. The proportion of patients with a pneumonia/LRTI was significantly associated with the age group (Chi sq (6) =37.9, p < 0.0001) but not the sex of the patient (Figure 2). As this figure demonstrates, the proportion of patients with a pneumonia/LRTI rose with increasing age and was highest among patients aged 80 years or more, where there were 26 patients (21.1%) with pneumonia. 

**Figure 3:**
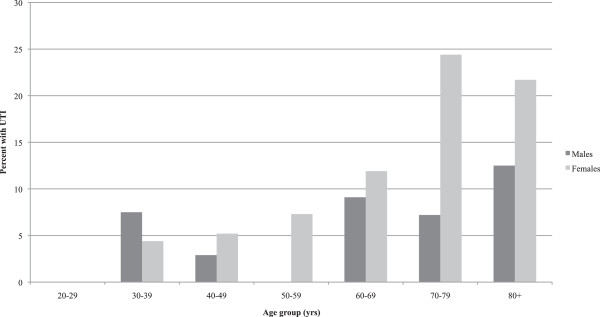
**Adrenal Crisis Admissions in NSW, 2000/1-2010/11.** Percentage with UTI by Age and Sex.

There were 82 admissions (10.0%) in which a UTI was identified. Of these, 64 (78.0%) were in women. As Figure 2 shows, the proportion of admissions with a UTI increased substantially with the age of the patient. Over one fifth of the women in both the 70–79 year age group (24.4%) and the 80 years and over age group (21.7%) had a UTI in association with the AC (Figure 3).

## Discussion

The results of the present study demonstrate that, while hypoadrenalism is rare in the general population, potentially life-threatening and largely preventable ACs occur relatively frequently. The study also highlights the important role that infection, especially bacterial infection, plays in hospital admissions among patients with hypoadrenalism who experience an AC, as has been found elsewhere [1, 2, 15, 16]. We found that older adults have a higher rate of AC-related admission than younger patients, and that the relative importance of other health problems, particularly infections, which occurred in association with the AC, varied with the age of the patient. In this study, we found that women represented approximately 60% of the total AC-related patient admissions, which is consistent with the male/female ratio seen in a Swedish study [17] and may be a reflection of the underlying prevalence of AI in the population, rather than a difference in risk of AC between the sexes [18].

Studies in other populations of patients with primary hypoadrenalism, have shown that ACs occur at an average rate of approximately 10% per year [3, 9, 15]. In the present study, the underlying cause of hypoadrenalism was recorded in only a minority of patient records and we were unable to provide reliable estimates of the incidence of AC by the cause of hypoadrenalism. However it is known that SAI is approximately twice as common as PAI, but the rates of AC are higher in PAI. A recent study reported that 65% of PAI patients had experienced an AC and a smaller proportion (47%) of SAI patients reported an AC [15]. Recurrent AC was also more common among those with PAI in that patient group [15]. By comparison, we believe, from clinical experience, that ACs are relatively rare in patients receiving glucocorticoids for treatment of other health problems, probably due to some residual adrenocortical function [16]. However, the results of the present study indicate that the overall AC rate did not reflect the differences in the frequencies of ACs between the different age groups. Older patients in the study group had both higher overall numbers of admissions in which an AC was documented and had correspondingly higher AC-related admission rates than younger patients. In addition, the proportion of patients recorded as dying during the admission in this study was low, given the life-threatening nature of an AC, with less than 1% of patients whose main medical problem was the AC recorded as having died in hospital.

The impact of exposure to bacterial infections on patients with hypoadrenalism is twofold. First, a number of studies have demonstrated that hypoadrenal patients are at increased risk of bacterial infection [2, 17, 18]. Second, the consequences of a bacterial infection in patients with hypoadrenalism differ from those experienced by patients with normal adrenal function. This is because systemic bacterial infection provokes a powerful inflammatory cytokine response stimulating the hypothalamic-pituitary-adrenal axis to produce a state of hypercortisolism. This, in turn, acts to reduce inflammation and prevent tissue damage. However, in the presence of cortisol deficiency, as is the case in Addison's disease, the absence of an increase in serum cortisol levels leads to an unrestrained inflammatory response, which results both in tissue damage and systemic effects, such as hypotension/shock and multi-organ failure [19]. To prevent the onset of an AC, patients are educated to follow a protocol for sick days, which incorporates the ‘3×3 rule’, or tripling the GC dose for three days or for the duration of a mild illness, after which the usual GC dose can be resumed [16]. In case of diarrhoea or vomiting, when oral medication cannot be taken or may not be absorbed, it is necessary to seek medical attention for parenteral therapy (50 mg IV/IM hydrocortisone followed by 25 mg IV hydrocortisone eight-hourly). For severe illness such as myocardial infarction, pancreatitis or sepsis, a dose of 50 mg IV hydrocortisone six hourly should be administered until the condition stabilises [20]. If there is any delay in obtaining medical care for parenteral hydrocortisone, patients or their relatives may administer hydrocortisone 100 mg IM from a self-injector kit. In addition, fludrocortisone, if taken for PAI, does not need dose adjustment, as hydrocortisone doses above 50 mg daily provide adequate mineralocorticoid cover.

Bacterial infection was a major contributing factor in the occurrence of the ACs in the patients in this study, and its frequency increased with the age of the patient. Pneumonia/LRTI and UTIs played a particularly important role, as has been shown in other populations [17, 18]. By comparison, gastroenteritis was present in fewer patients in this population with ACs than expected from other studies [9, 15]. Symptoms of acute hypoadrenalism, such as nausea and vomiting, overlap with those of gastroenteritis and may easily be misinterpreted by patients as acute gastroenteritis, potentially leading to an overestimation of the importance of infective gastroenteritis as a causal factor in the precipitation of an AC, especially in studies where patient self-reports are used as a data source. By comparison, hospital data, such as those used in this study, rely on the clinical diagnosis of medical professionals who are more likely to correctly differentiate between patients with hypoadrenalism who have gastroenteritis but not an AC and those who have nausea and vomiting as part of an AC.

This study highlights the importance of aging in the management of the hypoadrenal patient. Older patients with AI are at risk of a number of health problems such as cardiovascular disease, osteoporosis and infections [1, 17, 21, 22]. A predisposition to infection, together with the importance of bacterial infection in the initiation of an AC, suggests that a low threshold for instituting antibiotic therapy in older patients with AI may be warranted. Urinary tract infection was identified in 10% of the study sample, predominantly among women. Asymptomatic bacteriuria is common in older women [23] and, if these results are reproduced in further studies, recommendations for preventive strategies such as routine urine screening may be warranted and may prove to be of benefit in these patients.

In addition, there are particular characteristics of older patients that may make pharmacological management of hypoadrenalism more problematic. These include changes in the metabolism of pharmaceutical agents with age; drug interactions from the co-administration of multiple therapies; cognitive impairments resulting in issues with compliance [24] and problems with the management of stress-dosing when required [10]; and an increasing prevalence of co-morbid conditions with advancing age [24]. In addition, older patients may not experience typical symptoms of infection, such as fever, that would indicate a need for the administration of glucocorticoid stress doses. Moreover, in the elderly, the adverse effects of sepsis may be compounded by increased levels of confusion, making self-management more difficult.

Monitoring changes in the morbidity and mortality from rare diseases in populations is difficult. Examination of the incidence of ACs is one way of evaluating the health outcomes of patients with hypoadrenalism. Aggregated hospital data, such as those used in this study, are one source of information that may be useful in assessing changes over time and between groups. These data do not rely on patient self-report or on patient surveys, which frequently have problems with response rates and, therefore, are subject to selection bias. However, as we have shown in this study, data sources that rely on the identification of an AC as the principal diagnosis alone would under-represent the extent of the problem in a population. This is especially so in the oldest age groups because, as this study demonstrates, older patients are less likely to have the AC recorded as their principal diagnosis than younger patients.

While this administrative dataset comprises information on the hospital treatment given in NSW predominantly to residents of that state, it is possible that some patients received treatment interstate and vice-versa, although this is likely to represent a very small proportion of the patient group. In addition, these data deal with episodes of patient care and we were unable to identify whether there is a group of patients who have multiple admissions for the same problem, a phenomenon that has been demonstrated elsewhere among patients with hypoadrenalism [9, 15]. While the data are subjected to regular audits both at the hospital and also at the administrative departmental level, it is possible that some ACs are missed, resulting in an underestimation of the true AC rate. Further, where there was an admission to hospital with an extended LOS, the timeframe of the AC in the course of the patient’s illness could not be determined. Moreover, diagnostic certainty may differ between major teaching hospitals and smaller rural hospitals and between clinicians with varying levels of experience. In addition, these data are restricted to occurrences of illness where a patient was admitted to hospital. Episodes of acute AI managed successfully by the patient or by a medical practitioner in the community would not be included. Similarly, ACs which occurred out of hospital and which had a fatal outcome would not be identified. It is also possible that some patients in this dataset presented with an AC as their first manifestation of hypoadrenalism, although this was likely to be a small number of patients.

Self-management is the cornerstone of adrenal replacement therapy. Patient education sessions, with regular opportunities for reinforcement of key messages, are crucial elements in avoiding adverse outcomes. Ensuring adequate absorption of GC replacement therapy, stress dosing during periods of illness and parenteral administration of hydrocortisone, when necessary, are the essential components of the management of the patient with hypoadrenalism. Optimal management necessitates effective communication between patient and health care professionals. However, evidence from a number of studies in different populations demonstrates that many patients lack the capacity to manage episodes of acute AI effectively [10–12]. The results of the present study highlight the need for ongoing emphasis on AC prevention in the clinical care of patients with hypoadrenalism, especially among older patients.

## Conclusion

The incidence of AC increases with the increasing age of the patient. Infections, especially bacterial infections, are frequently associated with an AC and this association increases with the increasing age of the patient.
